# Effects of subcutaneous or oral semaglutide on cardiovascular outcomes in patients with type 2 diabetes mellitus: a meta-analysis of randomized controlled trials

**DOI:** 10.3389/fcvm.2025.1731127

**Published:** 2025-12-15

**Authors:** Sihua Tan, Yangguang Yin, Juexiu Lu

**Affiliations:** Department of Cardiology, The First Affiliated Hospital of Chongqing Medical and Pharmaceutical College, Chongqing, China

**Keywords:** cardiovascular disease, major adverse cardiovascular events, semaglutide, type 2 diabetes mellitus, meta-analysis

## Abstract

**Background:**

Glucagon-like peptide−1 receptor agonists (GLP-1 RAs), such as semaglutide, reduce cardiovascular risk in type 2 diabetes mellitus (T2DM), but the consistency between oral and subcutaneous formulations remains unclear.

**Methods:**

This meta-analysis was registered prospectively in PROSPERO (CRD 420251147337). A systematic search of PubMed, Embase, Cochrane Library, and Web of Science identified randomized controlled trials (RCTs) on semaglutide and cardiovascular outcomes. Pooled hazard ratios (HRs) with 95% confidence intervals (CIs) were calculated using fixed-/random-effects models, with sensitivity, subgroup, and GRADE assessments.

**Results:**

Four RCTs (*n* = 19,663) showed semaglutide significantly reduced primary outcome risk (HR 0.83; 95% CI 0.76–0.91), nonfatal myocardial infarction (HR 0.79; 0.67–0.92), and revascularization (HR 0.71; 0.61–0.83), with a modest decrease in heart failure hospitalization (HR 0.85; 0.72–1.00). No significant effects were seen for cardiovascular death, all-cause death, nonfatal stroke, or unstable angina hospitalization. Subgroup analyses confirmed no efficacy differences between formulations. Evidence quality was “moderate” for cardiovascular death, all-cause death, nonfatal stroke, unstable angina hospitalization, and “high” for the remainder.

**Conclusions:**

Semaglutide lowers cardiovascular risk in T2DM, primarily improving major adverse cardiovascular events, nonfatal myocardial infarction, and revascularization, with oral and subcutaneous forms demonstrating consistent efficacy.

**Systematic Review Registration:**

https://www.crd.york.ac.uk/PROSPERO/view/CRD420251147337, PROSPERO CRD420251147337.

## 1 Introduction

Type 2 diabetes mellitus (T2DM) is a prevalent chronic metabolic disorder primarily affecting middle-aged and elderly populations. However, driven by shifts in dietary patterns and rising obesity rates, its incidence and prevalence are steadily increasing, with the disease increasingly manifesting in younger individuals (1). Globally, an estimated 828 million adults live with diabetes, of whom over 90% have T2DM (1, 2). Cardiovascular diseases (CVDs), notably myocardial infarction, heart failure, and stroke, represent the leading causes of mortality in this patient group (3). Beyond stringent glycemic control, more effective interventions are imperative to reduce the risk of cardiovascular events in individuals with T2DM.

Glucagon-like peptide-1 receptor agonists (GLP-1 RAs) are a novel class of antidiabetic agents that activate the GLP-1 receptor to stimulate insulin secretion and inhibit glucagon secretion in a glucose-dependent manner, delay gastric emptying, thereby controlling blood glucose and promoting weight loss (4). Emerging evidence demonstrates that GLP-1 RAs, epitomized by semaglutide, exert established cardiovascular protective effects (5). This effect likely operates via multiple pathways: reducing key cardiovascular risk factors, including blood glucose, blood pressure, dyslipidemia, body weight, and inflammation, while directly inhibiting atherosclerotic progression and stabilizing plaques, ultimately improving atherosclerosis-related clinical outcomes (6). For injectable semaglutide, cardiovascular efficacy has been clearly validated in patients with T2DM who have concomitant CVD, high cardiovascular risk, or chronic kidney disease (CKD) (7–9). However, the consistency of efficacy between oral and injectable formulations remains a subject of debate (10, 11).

Previous meta-analyses have primarily pooled the cardiovascular effects of GLP-1 RAs in patients with T2DM (12–15), meanwhile only a few have evaluated the cardiovascular benefits of semaglutide specifically in individuals with overweight or obesity (16, 17). However, no recent meta-analysis has directly compared the impact of subcutaneous vs. oral semaglutide on cardiovascular outcomes, a critical gap that limits high-level evidence to guide clinical administration choices and improve patient treatment adherence.

This study performed a systematic review and meta-analysis to evaluate the effects of subcutaneous vs. oral semaglutide on cardiovascular outcomes in patients with T2DM. Using standardized literature screening, we included four randomized controlled trials (RCTs): SUSTAIN 6, PIONEER 6, FLOW, and SOUL. This work provides the first systematic assessment of subcutaneous vs. oral semaglutide's impact on cardiovascular outcomes in T2DM patients, aiming to clarify whether the two formulations demonstrate consistent efficacy in reducing cardiovascular events and mortality. That is critical for guiding clinical medication management, optimizing treatment strategies, and improving long-term prognosis in T2DM patients.

## 2 Methods

### 2.1 Eligibility criteria

This meta-analysis was registered prospectively in PROSPERO (CRD420251147337) and was carried out following PRISMA (Preferred Reporting Items for Systematic Reviews and Meta-Analyses) recommendations. The review included English-published RCTs with a control group, involving participants aged ≥18 years with T2DM that reported cardiovascular outcomes. Additionally, we excluded non-randomized studies, observational studies, case reports, reviews, letters to the editor, non-human studies, non-English articles, conference proceedings, and non-peer-reviewed articles.

### 2.2 Information sources and search strategy

A thorough literature search was carried out in PubMed, Embase, Cochrane Library, and Web of Science up to May 14, 2025. The descriptors used were (“type 2 diabetes” OR “T2DM”) AND (“glucagon-like peptide-1 receptor agonists” OR “semaglutide”) AND (“cardiovascular diseases” OR “major adverse cardiac events” OR “mortality” OR “stroke” OR “heart failure” OR “myocardial infarction”) AND (“randomized controlled trial” OR “RCT” OR “placebo”).

### 2.3 Literature screening, data extraction

All articles found in the data bases were exported to the Endnote platform. Initially, duplicates were removed and then titles and abstracts were read. Two researchers independently extracted data, resolving discrepancies through discussion or with input from a third author to achieve consensus. Extracted data included title, trial name, the first author, publication year, country/region, dose, administration route, median follow-up time, sample size, age, sex, race, body mass index (BMI), smoke, blood pressure, glycated hemoglobin (HbA1c), T2DM duration, estimated glomerular filtration rate (eGFR), baseline CVD or CKD, and measures of effect assessed by the hazard ratio (HR) and its 95% confidence interval (CI) for each of the cardiovascular outcomes.

### 2.4 Outcome definition

The primary outcome was defined as major adverse cardiovascular events (a three-point composite of death from cardiovascular causes, nonfatal myocardial infarction, or nonfatal stroke). The secondary outcomes included cardiovascular death, all-cause death, nonfatal myocardial infarction (MI), nonfatal stroke, heart failure (HF) hospitalization, unstable angina (UA) hospitalization, and revascularization.

### 2.5 Study risk of bias assessment

We evaluated the study quality using the Cochrane Collaboration's Risk of Bias tool (18). This tool assessed seven aspects: random sequence generation, allocation concealment, participant blinding, outcome assessment blinding, incomplete data handling, selective reporting, and other biases. The bias of the articles was classified as follows: low risk of bias, unclear risk of bias, and high risk of bias. Two authors independently evaluated the risk of bias, and any disagreements were resolved through discussion with a third author.

### 2.6 Data analysis

For each outcome, meta-analysis was performed using data pooled from at least two independent trials. Data synthesis was stratified by administration route and pooled concurrently. The outcomes synthesized included the HR with 95% CI for 3-point major adverse cardiovascular events (MACE), its individual components, all-cause death, HF hospitalization, UA hospitalization, and revascularization.

Heterogeneity across studies was evaluated using Cochran's *Q* test and the *I*^2^ statistic. Significant heterogeneity was defined as a *P*-value < 0.10 for Cochran's *Q* test or an *I*^2^ statistic exceeding 50%. When heterogeneity was significant, effect estimates were pooled using a random-effects model; when non-significant, a fixed-effects model was employed.

Subsequently, sensitivity analysis was performed to assess the robustness of the results. Additionally, for the primary outcome, subgroup analysis was conducted based on predefined factors as follows: age, sex, BMI, race, and eGFR, to explore potential sources of heterogeneity. *P* for interaction<0.05 indicates statistically significant differences between subgroups. Both data synthesis and sensitivity analysis were performed using RevMan version 5.4 and Stata version 15.0.

Finally, to detect potential publication bias, funnel plots and Egger's test were conducted. All analyses were independently executed by two researchers, with any disagreements resolved through consensus. Funnel plots were used for visual inspection, and Egger's test was applied to outcomes with three or more studies. A *p*-value below 0.05 was interpreted as evidence of significant publication bias. Due to only two studies meeting the Revascularization outcome criteria, quantitative sensitivity analysis and Egger's test were not performed; instead, qualitative descriptive comparison was employed.

### 2.7 GRADE assessment

The GRADE approach was applied to assess the overall confidence in the effect estimates for the primary and secondary outcomes. Factors such as study quality, consistency of results, and directness of evidence were considered. Certainty was classified as high, moderate, low, or very low based on predefined GRADE criteria (19).

## 3 Results

### 3.1 Literature search and study characteristics

**Figure 1:**
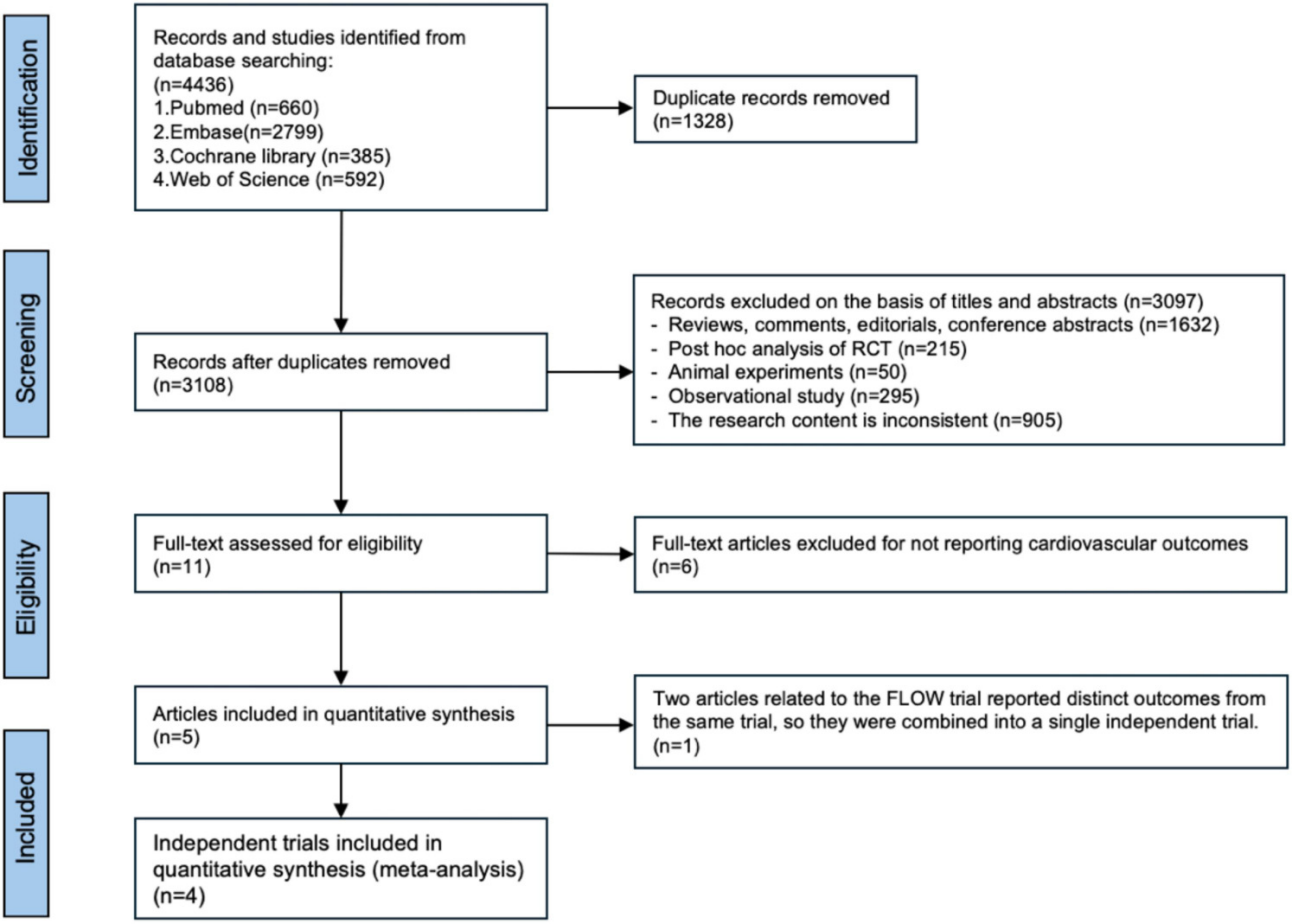
Flowchart of selection process.

The flowchart detailing the systematic literature search and selection process was presented in Figure 1. The 4,436 articles found were distributed as follows: 660 were found in the PubMed, 2,799 in the Embase, 385 in the Cochrane Library, 592 in the Web of Science data bases. The number of duplicates excluded was 1,328, resulting in 3,108 articles. Out of those, 3,097 were excluded after the title and abstracts were read. Therefore, 11 full texts were read. After reading the full article, 6 were excluded for not reporting cardiovascular outcomes. Notably, two articles related to the FLOW trial reported distinct outcomes from the same trial, so they were combined into a single independent trial. Finally, 4 studies were included in the meta-analysis (8–11, 20).

Tables 1, 2 presented the characteristics of the included studies and patient populations. All 4 studies were multicenter, randomized, double-blind, placebo-controlled trials, encompassing 19,663 patients in total (semaglutide group: 9,831; control group: 9,832). Sample sizes ranged from 3,183 to 9,650 patients, with the average age of participants between 64.6 and 66.6 years. Among the 4 included studies, 2 employed subcutaneous injection, while the remaining 2 used oral administration. Median follow-up durations ranged from 1.3 to 4.1 years. The proportion of male participants varied from 60.7% to 71.1%, and the proportion of participants with baseline CVD or CKD history ranged from 82.9% to 100%. Additionally, the proportion of participants with eGFR <60 mL/min/1.73 m^2^ varied from 28.5% to 79.6%, while the proportion of participants with BMI ≤ 30 kg/m^2^ fluctuated between 35.8% and 47.7%. The HbA1c values alternated between 7.8% and 8.7%. Finally, the background medications used in the four included studies were all standard treatments.

**Table 1:** Baseline characteristics of the included studies.

Trial name	Public-ation year	Country/region	Sample size (*n*)	Administr-ation route	Dose	Median follow-up time	Baseline CVD or CKD (*n*, %)	eGFR < 60 mL/min/1.73 m^2^ (*n*, %)
SUSTAIN 6	2016	at 230 sites in 20 countries	3,297	subcutaneous	0.5/ 1.0 mg weekly	2.1 year	2,735 (82.9%)	939 (28.5%)
PIONEER 6	2019	at 214 sites in 21 countries	3,183	oral	14 mg daily	1.3 year	2,695 (84.7%)	856 (26.9%)
FLOW	2024	at 387 sites in 28 countries	3,533	subcutaneous	1.0 mg weekly	3.4 year	3,533 (100%)	2,813 (79.6%)
SOUL	2025	at 444 sites in 33 countries	9,650	oral	14 mg daily	4.1 year	9,329 (96.7%)	2,816 (29.2%)

CVD, cardiovascular disease; CKD, chronic kidney disease; eGFR, estimated glomerular filtration rate.

**Table 2:** Patients’ demographic and characteristics.

Trial name	Sample data I/C (*n*)	Age (mean ± SD)	Male (*n*, %)	BMI ≤ 30 kg/m² (n,%)	HbA1c	Primary outcome I/C(*n*)	Cardiovascular causes I/C (*n*)	Nonfatal MI I/C (*n*)	Nonfatal stroke I/C (*n*)	All-cause death I/C (*n*)	HF hospitalization I/C (*n*)	UA hospitalization I/C (*n*)	Revascularization I/C (*n*)
SUSTAIN 6	1,648/1,649	64.6 ± 7.4	2,002 (60.7%)	1,180 (35.8%)	8.7 ± 1.5	108/146	44/46	47/64	27/44	62/60	59/54	22/27	83/126
PIONEER 6	1,591/1,592	66.0 ± 7.0	2,176 (68.4%)	1,279 (40.2%)	8.2 ± 1.6	61/76	15/30	37/31	12/16	23/45	21/24	11/7	NA
FLOW	1,767/1,766	66.6 ± 9.0	2,464 (69.7%)	1,467 (41.5%)	7.8 ± 1.3	212/254	123/169	52/64	63/51	227/279	133/175	N/A	NA
SOUL	4,825/4,825	66.1 ± 7.5	6,860 (71.1%)	4,602 (47.7%)	8.0 ± 1.2	579/668	301/320	191/253	144/161	528/577	146/167	74/80	200/263

I, intervention group; C, control group; BMI: body mass index; HbA1c, glycated hemoglobin; SD, standard deviation; MI, myocardial infarction; HF, heart failure; UA, unstable angina.

### 3.2 Study risk of bias assessment

**Figure 2:**
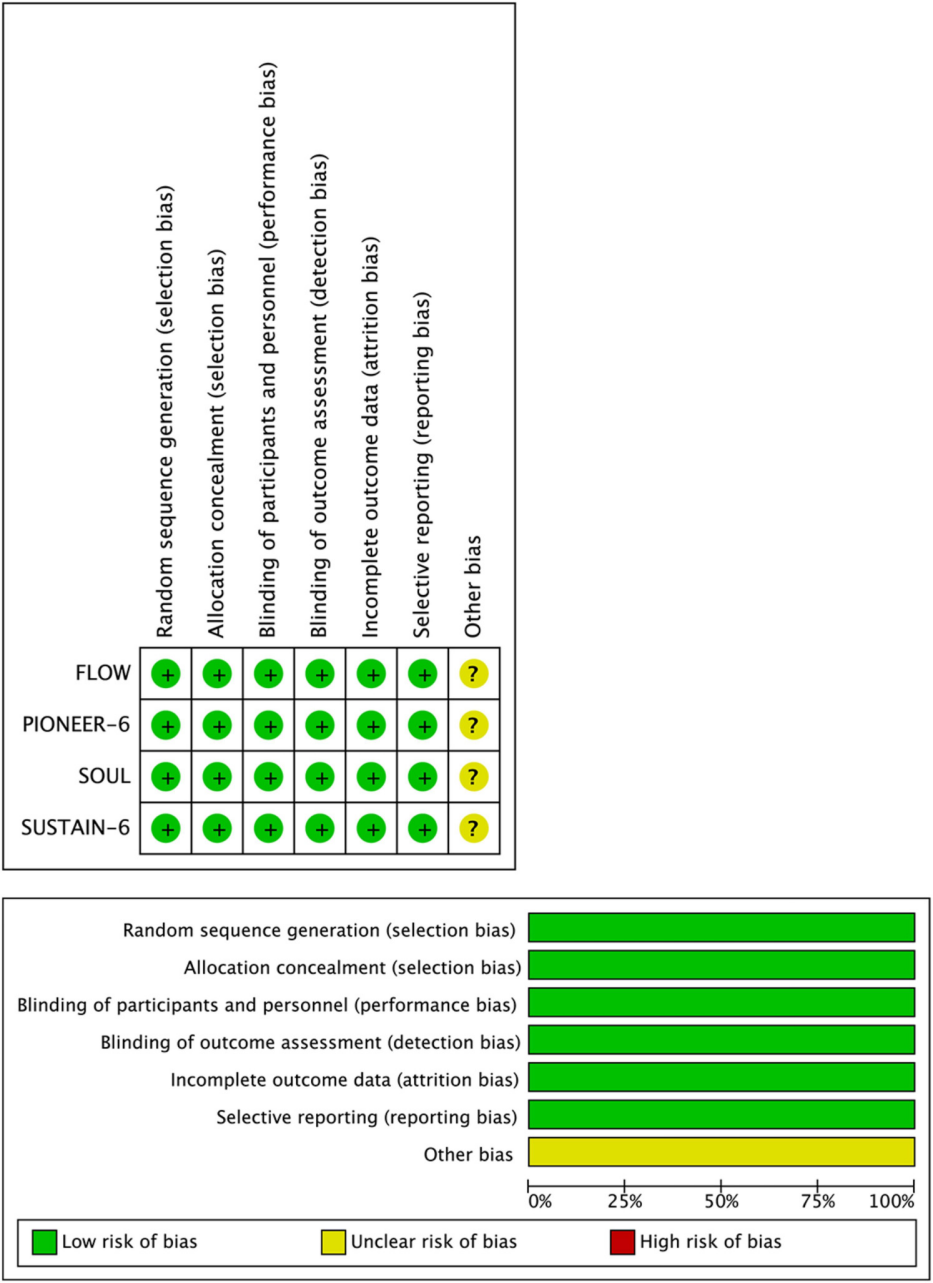
Risk of bias assessment for included RCTs.

The risk of bias in all included studies was assessed using the Cochrane Collaboration's Risk of Bias tool. The assessment results indicated that all studies had low risk of bias in key domains, including random sequence generation, allocation concealment, blinding, detection bias, incomplete outcome data, and selective reporting, whereas other sources of bias were unclear. Overall, the methodological quality of the four studies was rated as high (Figure 2).

### 3.3 Primary outcome

**Figure 3:**
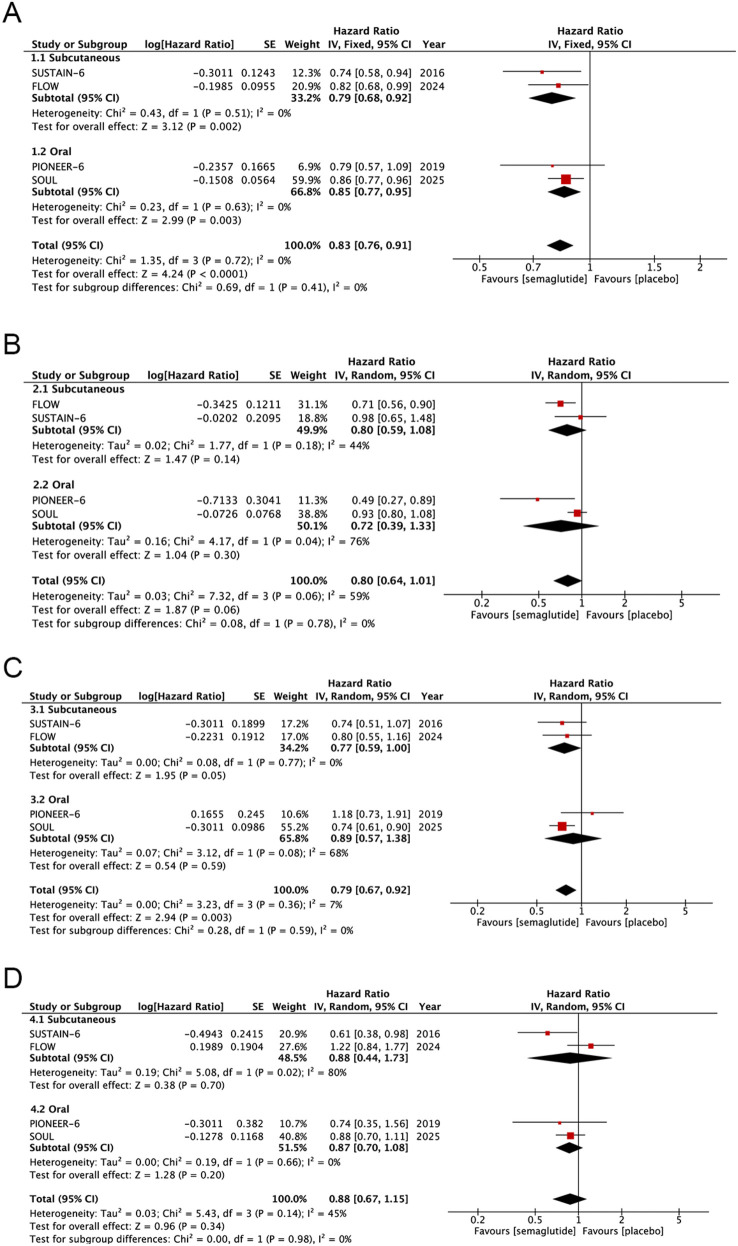
Forest plot of pooled HRs for cardiovascular outcomes: **(A)** primary outcome, **(B)** cardiovascular death, **(C)** nonfatal myocardial infarction, and **(D)** nonfatal stroke.

We analyzed four studies involving a total of 19,663 patients to evaluate the effect of semaglutide on the primary outcome, and specifically to determine whether there were significant differences in the impact of subcutaneous vs. oral semaglutide formulations on the primary outcome. During follow-up, 960 of 9,831 (9.8%) patients in the semaglutide group experienced the primary outcome, compared with 1,144 of 9,832 (11.6%) patients in the control group. The forest plot (Figure 3A) displays the HR and its 95% CI for each individual study, as well as the pooled HR and 95% CI stratified by administration route (subcutaneous vs. oral). Pooled analysis of the 4 studies demonstrated that semaglutide significantly reduced the risk of the primary outcome (HR: 0.83; 95% CI: 0.76–0.91; *P* < 0.0001), with no significant heterogeneity observed across studies (*I*^2^ = 0%, *P* = 0.72). Furthermore, subgroup difference analysis revealed no statistically significant discrepancy in outcomes between the two administration routes (*I*^2^ = 0%, *P* = 0.41). Assessment using the funnel plot (Supplementary Figure S1A) and Egger's test indicated no significant publication bias (*P* = 0.145).

### 3.4 Secondary outcomes

#### 3.4.1 Cardiovascular death

Four studies including 19,663 patients were analyzed to evaluate semaglutide's effect on cardiovascular death and differences between subcutaneous and oral semaglutide. During follow-up, 483 of 9,831 (4.9%) patients in the semaglutide group experienced cardiovascular death, compared with 565 of 9,832 (5.7%) patients in the control group. The forest plot (Figure 3B) displays all the analysis results. Pooled analysis demonstrated that semaglutide did not significantly reduce the risk of cardiovascular death compared with placebo (HR: 0.80; 95% CI: 0.64–1.01; *P* = 0.06), with significant heterogeneity observed across studies (*I*^2^ = 59%, *P* = 0.06). Furthermore, subgroup difference analysis revealed no statistically significant discrepancy in outcomes between the two administration routes (*I*^2^ = 0%, *P* = 0.78). Assessment using the funnel plot (Supplementary Figure S1B) and Egger's test indicated no significant publication bias (*P* = 0.429).

#### 3.4.2 Nonfatal myocardial infarction

Four studies including 19,663 patients were analyzed to evaluate semaglutide's effect on nonfatal myocardial infarction and differences between subcutaneous and oral semaglutide. During follow-up, 327 of 9,831 (3.3%) patients in the semaglutide group experienced nonfatal myocardial infarction, compared with 412 of 9,832 (4.2%) patients in the control group. The forest plot (Figure 3C) displays all the analysis results. Pooled analysis demonstrated that semaglutide significantly reduced the risk of nonfatal myocardial infarction compared with placebo (HR: 0.79; 95% CI: 0.67–0.92; *P* = 0.003), with no significant heterogeneity observed across studies (*I*^2^ = 7%, *P* = 0.36). Furthermore, subgroup difference analysis revealed no statistically significant discrepancy in outcomes between the two administration routes (*I*^2^ = 0%, *P* = 0.59). Assessment using the funnel plot (Supplementary Figure S1C) and Egger's test indicated no significant publication bias (*P* = 0.288).

#### 3.4.3 Nonfatal stroke

Four studies including 19,663 patients were analyzed to evaluate semaglutide's effect on nonfatal stroke and differences between subcutaneous and oral semaglutide. During follow-up, 246 of 9,831 (2.5%) patients in the semaglutide group experienced nonfatal stroke, compared with 272 of 9,832 (2.8%) patients in the control group. The forest plot (Figure 3D) displays all the analysis results. Pooled analysis demonstrated that semaglutide did not significantly reduce the risk of nonfatal stroke compared with placebo (HR: 0.88; 95% CI: 0.67–1.15; *P* = 0.34), with no significant heterogeneity observed across studies (*I*^2^ = 45%, *P* = 0.14). In addition, subgroup difference analysis revealed no statistically significant discrepancy in outcomes between the two administration routes (*I*^2^ = 0%, *P* = 0.98). Assessment using the funnel plot (Supplementary Figure S1D) and Egger's test indicated no significant publication bias (*P* = 0.714).

#### 3.4.4 All-cause death

**Figure 4:**
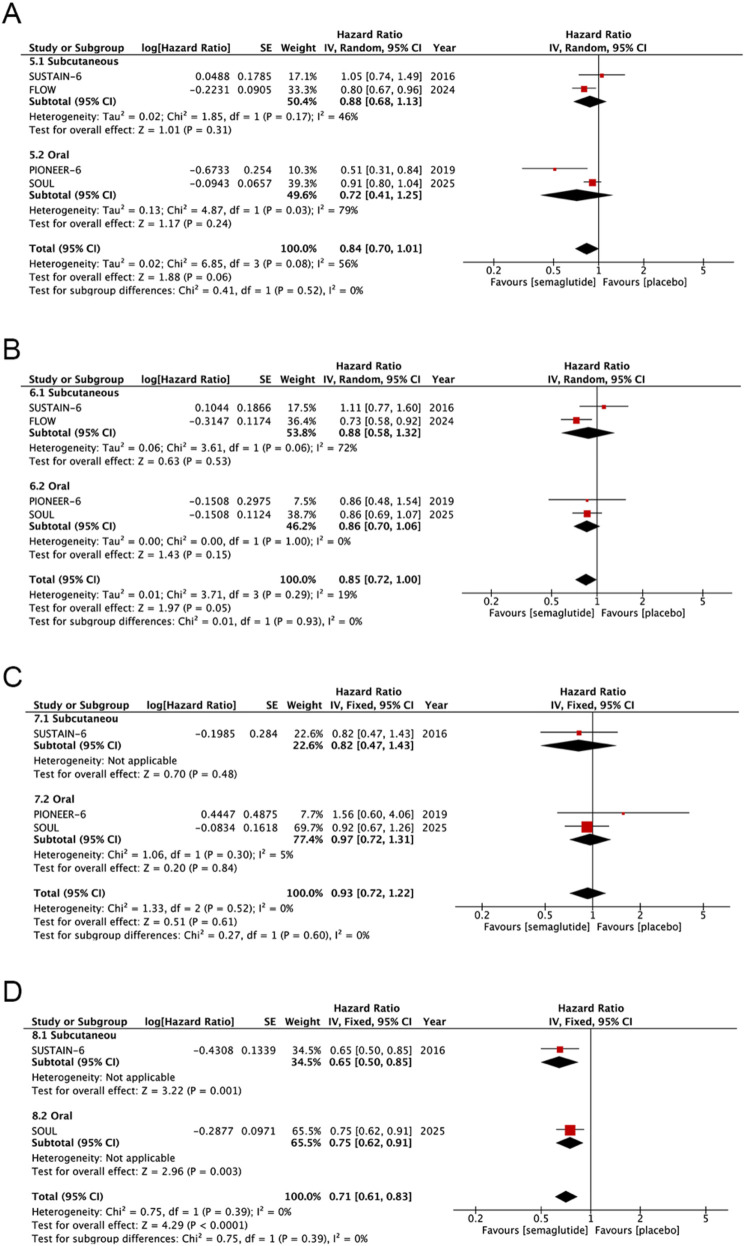
Forest plot of pooled HRs for cardiovascular outcomes: **(A)** All-cause death, **(B)** HF hospitalization, **(C)** UA hospitalization, and **(D)** revascularization.

Four studies including 19,663 patients were analyzed to evaluate semaglutide's effect on all-cause death and differences between subcutaneous and oral semaglutide. During follow-up, 840 of 9,831 (8.5%) patients in the semaglutide group experienced all-cause death, compared with 961 of 9,832 (9.8%) patients in the control group. The forest plot (Figure 4A) shows all the analysis results. Pooled analysis demonstrated that semaglutide did not significantly reduce the risk of all-cause death compared with placebo (HR: 0.84; 95% CI: 0.70–1.01; *P* = 0.06), with significant heterogeneity observed across studies (*I*^2^ = 56%, *P* = 0.08). In addition, subgroup difference analysis revealed no statistically significant discrepancy in outcomes between the two administration routes (*I*^2^ = 0%, *P* = 0.52). Assessment using the funnel plot (Supplementary Figure S2A) and Egger's test indicated no significant publication bias (*P* = 0.525).

#### 3.4.5 HF hospitalization

Four studies including 19,663 patients were analyzed to evaluate semaglutide's effect on HF hospitalization and differences between subcutaneous and oral semaglutide. During follow-up, 359 of 9,831 (3.7%) patients in the semaglutide group experienced HF hospitalization, compared with 420 of 9,832 (4.3%) patients in the control group. The forest plot (Figure 4B) shows all the analysis results. Pooled analysis demonstrated that semaglutide did not significantly reduce the risk of HF hospitalization compared with placebo (HR: 0.85; 95% CI: 0.72–1.00; *P* = 0.05), with no significant heterogeneity observed across studies (*I*^2^ = 19%, *P* = 0.29). In addition, subgroup difference analysis revealed no statistically significant discrepancy in outcomes between the two administration routes (*I*^2^ = 0%, *P* = 0.93). Assessment using the funnel plot (Supplementary Figure S2B) and Egger's test indicated no significant publication bias (*P* = 0.524).

#### 3.4.6 UA hospitalization

Three studies including 16,130 patients were analyzed to evaluate semaglutide's effect on UA hospitalization and differences between subcutaneous and oral semaglutide. During follow-up, 107 of 8,064 (1.3%) patients in the semaglutide group experienced UA hospitalization, compared with 114 of 8,066 (1.4%) patients in the control group. The forest plot (Figure 4C) shows all the analysis results. Pooled analysis demonstrated that semaglutide did not significantly reduce the risk of UA hospitalization compared with placebo (HR: 0.93; 95% CI: 0.72–1.22; *P* = 0.61), with no significant heterogeneity observed across studies (*I*^2^ = 0%, *P* = 0.52). Additionally, subgroup difference analysis revealed no statistically significant discrepancy in outcomes between the two administration routes (*I*^2^ = 0%, *P* = 0.60). Assessment using the funnel plot (Supplementary Figure S2C) and Egger's test indicated no significant publication bias (*P* = 0.564).

#### 3.4.7 Revascularization

Two studies including 12,947 patients were analyzed to evaluate semaglutide's effect on revascularization and differences between subcutaneous and oral semaglutide. During follow-up, 283 of 6,473 (4.4%) patients in the semaglutide group experienced revascularization, compared with 389 of 6,474 (6.0%) patients in the control group. The forest plot (Figure 4D) shows all the analysis results. Pooled analysis demonstrated that semaglutide significantly reduced the risk of revascularization compared with placebo (HR: 0.71; 95% CI: 0.61–0.83; *P* = 0.61), with no significant heterogeneity observed across studies (*I*^2^ = 0%, *P* = 0.39). Additionally, subgroup difference analysis revealed no statistically significant discrepancy in outcomes between the two administration routes (*I*^2^ = 0%, *P* = 0.39). The funnel plot (Supplementary Figure S2D) showed evenly distributed points. Combined with ROB2 bias risk assessment, both studies were large-sample, high-quality RCTs with significant benefits and no negative results, so there was no significant publication bias.

### 3.5 Subgroup analysis

The results of subgroup analyses were summarized in Table 3. Based on five pre-defined factors potentially contributing to heterogeneity, we conducted a subgroup analysis for the primary outcome. Given that the primary outcome of the FLOW trial was major kidney disease events rather than 3-point MACE, the FLOW trial was excluded from this subgroup analysis. In the subgroup analysis, semaglutide exhibited no statistical heterogeneity in its effects on patients aged <60 years vs. ≥65 years [HR: 0.76 (95% CI: 0.65–0.89) vs. HR: 0.87 (95% CI: 0.77–0.98)], indicating no significant interaction effect (*P* = 0.18). Nor was there any interaction (*P* = 0.70) when stratifying trials by sex. Similarly, subgroup analyses based on BMI (≤30 kg/m^2^ vs. >30 kg/m^2^), race (White vs. Asian vs. Black vs. Other), and eGFR (<60 mL/min/1.73 m^2^ vs. ≥60 mL/min/1.73 m^2^) revealed no statistical heterogeneity in semaglutide's efficacy for reducing MACE risk.

**Table 3:** Subgroup analysis of primary outcome.

Subgroup	Primary outcome			
	No. of study	HR [95%CI]	*I*²	*P* value for interaction
Age				
<65 year	3	0.76 (0.65, 0.89)	10%	0.18
≥65 year	3	0.87 (0.77, 0.98)	0%	
Sex				
Male	3	0.81 (0.73, 0.91)	9%	0.70
Female	3	0.85 (0.70, 1.04)	0%	
BMI				
≤30 kg/m²	3	0.72 (0.54, 0.95)	50%	0.28
>30 kg/m²	3	0.85 (0.74, 0.97)	0%	
Race				
White	3	0.87 (0.78, 0.97)	0%	0.17
Asian	3	0.70 (0.60, 0.99)	22%	
Black	3	0.88 (0.31, 2.47)	52%	
Other	3	0.51 (0.30, 0.85)	0%	
eGFR				
<60 mL/min/1.73 m²	3	0.80 (0.68, 0.95)	0%	0.68
≥60 mL/min/1.73 m²	3	0.84 (0.74, 0.95)	8%	

No., number; HR, hazard ratio; CI: confidence interval; BMI, body mass index; eGFR, estimated glomerular filtration rate.

### 3.6 Sensitivity analysis

Sensitivity analyses were conducted for each cardiovascular outcome to assess the influence of individual studies on the pooled HRs. This was achieved by excluding one study at a time and observing the changes in the pooled HRs. Sensitivity analysis reveals the degree of robustness of the results as follows (Supplementary Figures S3, S4):

i) **Primary outcome & secondary outcomes (nonfatal stroke, all-cause death, HF hospitalization):** Excluding any single study did not alter the direction and magnitude of effects, confirming robustness.
ii) **Cardiovascular death:** Excluding the SOUL trial strengthened the effect (shifting from non-significant to significant), suggesting sensitivity to SOUL but moderate robustness due to consistent overall effect direction and magnitude.
iii) **Nonfatal myocardial infarction:** Excluding any study did not change effects; however, excluding PIONEER 6 enhanced benefit and narrowed the CI, indicating greater robustness post-exclusion.
iv) **UA hospitalization:** Excluding SOUL reversed the effect direction, signaling sensitivity to SOUL and lower robustness.
v) **Revascularization:** With only two studies, descriptive comparison showed: removing SUSTAIN 6 yielded a consistent HR direction [0.75 (0.62–0.90) vs. pooled 0.71 (0.61–0.83)]; fixed- and random-effects models both gave HR = 0.71 (difference <0.01), confirming model insensitivity and robustness.

### 3.7 GRADE assessment

In the GRADE assessment, the HR effects for nonfatal stroke and UA hospitalization were downgraded to “moderate” due to imprecision [no statistically significant results and total event counts below the optimal information size (OIS) threshold]. Additionally, cardiovascular death and all-cause death were also rated “moderate” due to significant statistical heterogeneity that could not be explained by subgroup analysis (inconsistency). For other cardiovascular outcomes—including the primary outcome, nonfatal myocardial infarction, HF hospitalization, and revascularization—no serious risks of bias, inconsistency, indirectness, or imprecision were identified, nor was publication bias detected. Reasonable confounding factors did not significantly affect the validity of the results, leading to a “high” quality of evidence rating for these outcomes (Table 4).

**Table 4:** GRADE evidence profile.

Outcome	No. of studies	Risk of bias	Inconsistency	Indirectness	Imprecision	Publication bias	Plausible confounding	Magnitude of effect	Dose-response gradient	Quality
Primary outcome	4	no serious risk	no serious inconsistency	no serious indirectness	no serious imprecision	undetected	would not reduce effect	no	no	High
Cardiovascular death	4	no serious risk	serious inconsistency	no serious indirectness	no serious imprecision	undetected	would not reduce effect	no	no	Moderate
Nonfatal MI	4	no serious risk	no serious inconsistency	no serious indirectness	no serious imprecision	undetected	would not reduce effect	no	no	High
Nonfatal stroke	4	no serious risk	no serious inconsistency	no serious indirectness	serious imprecision	undetected	would not reduce effect	no	no	Moderate
All-cause death	4	no serious risk	serious inconsistency	no serious indirectness	no serious imprecision	undetected	would not reduce effect	no	no	Moderate
HF hospitalization	4	no serious risk	no serious inconsistency	no serious indirectness	no serious imprecision	undetected	would not reduce effect	no	no	High
UA hospitalization	3	no serious risk	no serious inconsistency	no serious indirectness	serious imprecision	undetected	would not reduce effect	no	no	Moderate
Revascularization	2	no serious risk	no serious inconsistency	no serious indirectness	no serious imprecision	undetected	would not reduce effect	no	no	High

NO., number; MI, myocardial infarction; HF, heart failure; UA, unstable angina.

## 4 Discussion

Based on current RCT evidence, our meta-analysis showed that semaglutide significantly reduces MACE risk in patients with T2DM, primarily by lowering nonfatal myocardial infarction risk, with no significant effects on cardiovascular death or nonfatal stroke. Additionally, semaglutide significantly reduced revascularization risk. While its protective effects on all-cause death and HF hospitalization did not reach significance, potential benefits were suggested, particularly for reducing HF hospitalization risk. However, current evidence does not support its role in protecting UA hospitalization. Critically, subgroup analyses comparing subcutaneous vs. oral administration revealed no significant differences in cardiovascular efficacy between the two formulations. To our knowledge, this is the first meta-analysis systematically evaluating efficacy differences of subcutaneous vs. oral semaglutide on cardiovascular outcomes in T2DM patients, an advance with important clinical value. Sensitivity analyses further confirmed results robustness, except for UA hospitalization. Using GRADE criteria, evidence quality was “moderate” for four outcomes: cardiovascular death, all-cause death, nonfatal stroke, and UA hospitalization. The remaining four outcomes were rated “high”.

Compared with previous studies, our analysis not only reaffirms semaglutide's cardiovascular protective effects in patients with T2DM (21), especially those with comorbid CVD or CKD, but also provides the first evidence that subcutaneous and oral semaglutide formulations have consistent efficacy in this population. Cleto et al. (16) evaluated semaglutide's cardiovascular effects and safety in overweight/obese individuals via meta-analysis and found it reduced risks of HF hospitalization, cardiovascular death, all-cause death, nonfatal myocardial infarction, and coronary revascularization. The subcutaneous route was deemed more effective for preventing these outcomes. Additionally, a meta-analysis by Hosseinpour et al. (17) including GLP-1 RAs studies such as semaglutide showed GLP-1 RAs outperformed placebo for MACE, all-cause death, cardiovascular death, myocardial infarction, stroke, and HF hospitalization. Moreover, GLP-1 RAs had similar efficacy in overweight/obese non-diabetic patients vs. those with diabetes. A separate meta-analysis on semaglutide and heart failure linked its use to reduced heart failure-related clinical events, though heterogeneity existed across study populations (22). However, these studies did not address whether different semaglutide administration routes lead to differential cardiovascular effects in T2DM patients. Our analysis fills this gap by providing robust clinical evidence to guide formulation selection.

Our study is the first meta-analysis to evaluate semaglutide's impact on cardiovascular outcomes in patients with T2DM and comorbid CVD or CKD, while also comparing the efficacy of subcutaneous vs. oral formulations. Results showed that for the primary outcome, semaglutide significantly reduced MACE risk by 17%, consistent with effect sizes integrated from prior GLP-1 RAs meta-analyses (14), Heterogeneity tests indicated no statistical heterogeneity across studies, and subgroup analyses revealed no interactions, suggesting semaglutide's effect on MACE was unaffected by potential confounders such as age, sex, BMI, race, or eGFR. For cardiovascular death, heterogeneity tests indicated significant inter-study variability. Sensitivity analyses further showed results were sensitive to the SOUL trial. This heterogeneity may stem from differences in follow-up duration: short-term studies might overestimate effect sizes due to insufficient event accumulation, whereas long-term follow-up better supports conclusion stability. These findings suggest semaglutide may offer potential benefits for cardiovascular death in T2DM patients with CVD or CKD, though larger long-term RCTs are needed for confirmation. Similar patterns emerged for all-cause death, indicating semaglutide may reduce all-cause death risk in T2DM patients, which supports prioritized use in high-risk populations such as those with comorbid CVD or CKD. Additionally, our study demonstrated a significant 21% reduction in nonfatal myocardial infarction risk with semaglutide, accompanied by high evidence quality. This confirms semaglutide effectively prevents myocardial infarction in T2DM patients, positioning it as one of the optimal therapeutic agents for T2DM with atherosclerotic cardiovascular disease (ASCVD). For nonfatal stroke, our results suggested a potential weak protective effect of semaglutide in T2DM patients. However, due to limited event numbers, conclusions remained inconclusive, requiring larger long-term follow-up studies with bigger samples for validation. For heart failure hospitalization outcomes, the STEP-HFpEF DM trial (23) demonstrated that semaglutide significantly reduces heart failure-related symptoms and physical limitations, while Barbagelata et al. (22) also showed semaglutide use correlates with fewer heart failure-related clinical events. Our study revealed semaglutide's protective effect against HF hospitalization approached borderline statistical significance. This discrepancy may stem from our included populations having fewer baseline heart failure symptoms or related histories, yet this still supports semaglutide's high evidence certainty (GRADE high level) for reducing HF hospitalization risk in T2DM patients—particularly those with high-risk heart failure or CKD—endorsing its role as a core medication for heart failure prevention in T2DM. For unstable angina hospitalization, sensitivity analyses indicated non-robust results, suggesting current evidence is insufficient to support semaglutide's protective effect against this outcome; future research should focus more on this area. Finally, for revascularization, our study confirmed semaglutide significantly reduces revascularization risk (including coronary and peripheral artery) by 29%, aligning with findings from the STRIDE trial (24). That research first demonstrated semaglutide effectively improves lower extremity symptoms and exercise capacity in patients with symptomatic peripheral artery disease (PAD) and T2DM, further strengthening evidence for semaglutide's benefits across panvascular diseases. In summary, our results combined with these latest studies confirm semaglutide's protective effects across multiple clinical domains, supporting its status as a core medication for T2DM patients with atherosclerotic diseases or CKD.

GLP-1 RAs, exemplified by semaglutide, exert cardiovascular protection via multiple mechanisms (6, 25). First, they enhance insulin sensitivity, ameliorate insulin resistance, and lower blood glucose levels, thereby mitigating vascular damage caused by abnormal glucose metabolism—their most direct effect (26). Second, GLP-1 RAs delay gastric emptying and reduce food intake, significantly improving weight management in individuals with overweight or obesity and cutting CVD risk linked to metabolic disorders (26). Beyond these, GLP-1 RAs also have glucose- and weight-independent cardiovascular protective mechanisms: they regulate blood pressure, lower lipid levels, reduce chronic inflammation, improve endothelial function, stabilize plaques, increase coronary blood flow, and inhibit cardiomyocyte apoptosis and fibrosis (27). These mechanisms explain why GLP-1 RAs show potent cardiovascular protection in high-risk T2DM patients and provide evidence-based support for their use in this population.

Our findings hold significant clinical implications. This is the first comprehensive meta-analysis reporting differences in cardiovascular outcomes between T2DM patients treated with subcutaneous vs. oral semaglutide, and all included studies were high-quality cardiovascular outcome trials. Results support no significant differences in efficacy between subcutaneous and oral semaglutide formulations, suggesting clinicians select formulations based on patient adherence. It also recommends prioritizing its use in managing high-risk T2DM patients, especially those with comorbid ASCVD or CKD.

Our study has certain limitations. First, although all included studies were high-quality large-scale RCTs, their small number restricted our ability to explain sources of heterogeneity through subgroup analyses. Combined with sensitivity analyses, we infer that the imprecision leading to downgraded evidence quality for certain outcomes may be linked to differences in study follow-up duration: short-term follow-up studies might suffer from increased random error or insufficient statistical power due to inadequate accumulation of cardiovascular events. Thus, future research should incorporate more long-term follow-up RCTs to further validate these downgraded outcomes. Second, our analysis did not evaluate differences in adverse events between subcutaneous and oral semaglutide formulations. However, safety assessments of oral semaglutide in the SOUL trial (11) showed its overall safety profile aligned with prior semaglutide trials (28), with no new safety signals observed. Third, some included studies did not stratify results by key patient characteristics (e.g., insulin resistance levels, heart failure clinical phenotypes, cardiac function classification, or SGLT2 inhibitor use in background therapy), potentially limiting the relevance of findings to specific subgroups. Finally, our study population primarily focused on T2DM patients with comorbid CVD or CKD, restricting the generalizability of conclusions to general or low-risk T2DM populations. Additionally, the present study did not investigate renal outcome events. Future research should therefore specifically focus low-risk populations and comprehensively assess semaglutide's impact on cardiorenal outcomes.

## 5 Conclusion

Semaglutide significantly reduces cardiovascular risk in patients with T2DM, primarily by improving MACE, nonfatal myocardial infarction, and revascularization outcomes, while modestly lowering HF hospitalization risk. It is recommended to prioritize its use in patients with comorbid ASCVD or CKD. However, no significant reductions were observed in cardiovascular death, all-cause death, nonfatal stroke, or UA hospitalization risk. Additionally, subcutaneous and oral semaglutide formulations demonstrate consistent efficacy, supporting its role as a core medication for reducing cardiovascular risk in this population. Nevertheless, further long-term follow-up studies are needed to confirm its sustained effects and clarify its impact on mortality, stroke, unstable angina, and renal outcomes.

## Data Availability

The original contributions presented in the study are included in the article/Supplementary Material, further inquiries can be directed to the corresponding author.
