# Supplementary material for: Effects of subcutaneous or oral semaglutide on cardiovascular outcomes in patients with type 2 diabetes mellitus: a meta-analysis of randomized controlled trials
Source: Front Cardiovasc Med. 2025 Dec 15;12:1731127. doi: 10.3389/fcvm.2025.1731127 (PMC12745249; doi:10.3389/fcvm.2025.1731127)
Supplement: Supplementary file 1 [file Datasheet1.docx]

***Supplementary materials***

**Supplementary Table S1.** Literature Search Strategy

**Supplementary Figure S1.** Funnel Plot for Cardiovascular Outcomes: (A) Primary outcome, (B) Cardiovascular death, (C) Nonfatal myocardial infarction, and (D) Nonfatal stroke.

**Supplementary Figure S2.** Funnel Plot for Cardiovascular Outcomes: (A) All-cause death, (B) HF hospitalization, (C) UA hospitalization, and (D) Revascularization.

**Supplementary Figure S3.** Sensitivity Analysis of Pooled HRs for Cardiovascular Outcomes: (A) Primary outcome, (B) Cardiovascular death, (C) Nonfatal myocardial infarction, and (D) Nonfatal stroke.

**Supplementary Figure S4.** Sensitivity Analysis of Pooled HRs for Cardiovascular Outcomes: (A) All-cause death, (B) HF hospitalization, and (C) UA hospitalization.

**Table S1. Literature Search Strategy.**

1. **Pubmed**

| Search number | Query |
| --- | --- |
| #1 | "Diabetes Mellitus, Type 2"[Mesh] |
| #2 | "diabetes mellitus, type 2"[Title/Abstract] OR "Type 2 Diabetes"[Title/Abstract] OR "diabetes mellitus type 2"[Title/Abstract] OR "Noninsulin-Dependent Diabetes Mellitus"[Title/Abstract] OR "NIDDM"[Title/Abstract] OR "T2DM"[Title/Abstract] |
| #3 | #1 OR #2 |
| #4 | "Glucagon-Like Peptide-1 Receptor Agonists"[Mesh] |
| #5 | "Glucagon Like Peptide 1 Receptor Agonists"[Title/Abstract] OR "glp 1 receptor agonists"[Title/Abstract] OR "Incretin Mimetics"[Title/Abstract] OR "glp 1 analogs"[Title/Abstract] OR " semaglutide"[Title/Abstract] |
| #6 | #4 OR #5 |
| #7 | "Cardiovascular Diseases"[Mesh] |
| #8 | "Cardiovascular Diseases"[Title/Abstract] OR "Cardiac Events"[Title/Abstract] OR "Coronary Artery Disease"[Title/Abstract] OR "Major Adverse Cardiac Events"[Title/Abstract] OR "mortality"[Title/Abstract] OR "Myocardial Infarction"[Title/Abstract] OR "Heart Failure"[Title/Abstract] OR "Stroke"[Title/Abstract] |
| #9 | #7 OR #8 |
| #10 | "Randomized Controlled Trials as Topic"[Mesh] |
| #11 | "randomized controlled trial"[Title/Abstract] OR "Clinical Trials, Randomized"[Title/Abstract] OR "Trials, Randomized Clinical"[Title/Abstract] OR "Controlled Clinical Trials, Randomized"[Title/Abstract] OR "random*"[Title/Abstract] OR "placebo"[Title/Abstract] |
| #12 | #10 OR #11 |
| #13 | #3 AND #6 AND #9 AND #12 |

**2.Embase**

| Search number | Query |
| --- | --- |
| #1 | 'diabetes mellitus, type 2'/exp OR 'diabetes mellitus, type 2' |
| #2 | 'type 2 diabetes':ab,ti OR 'diabetes mellitus, type 2':ab,ti OR 'noninsulin-dependent diabetes mellitus':ab,ti OR 'niddm':ab,ti OR 't2dm':ab,ti |
| #3 | #1 OR #2 |
| #4 | 'glucagon like peptide 1 receptor agonist'/exp |
| #5 | 'glucagon like peptide 1 receptor agonists':ab,ti OR 'glp-1 receptor agonists':ab,ti OR 'glp 1 receptor agonists':ab,ti OR 'incretin mimetics':ab,ti OR 'glp-1 analogs':ab,ti OR 'semaglutide':ab,ti |
| #6 | #4 OR #5 |
| #7 | 'cardiovascular disease'/exp |
| #8 | 'cardiovascular disease':ab,ti OR 'cardiac events':ab,ti OR 'coronary artery disease':ab,ti OR 'major adverse cardiac events':ab,ti OR 'mortality':ab,ti OR 'myocardial infarction':ab,ti OR 'heart failure':ab,ti OR 'stroke':ab,ti |
| #9 | #7 OR #8 |
| #10 | 'randomized controlled trial'/exp |
| #11 | 'randomized controlled trial':ab,ti OR 'clinical trials, randomized':ab,ti OR 'trials, randomized clinical':ab,ti OR 'controlled clinical trials, randomized':ab,ti OR 'random*':ab,ti OR 'placebo':ab,ti |
| #12 | #10 OR #11 |
| #13 | #3 AND #6 AND #9 AND #12 |

**3.The Cochrane Library**

| Search number | Query |
| --- | --- |
| #1 | MeSH descriptor: [Diabetes Mellitus, Type 2] explode all trees |
| #2 | (Type 2 Diabetes):ti,ab,kw OR (Diabetes Mellitus, Type 2):ti,ab,kw OR (Noninsulin-Dependent Diabetes Mellitus):ti,ab,kw OR (NIDDM):ti,ab,kw OR (T2DM):ti,ab,kw |
| #3 | #1 OR #2 |
| #4 | MeSH descriptor: [Glucagon-Like Peptide-1 Receptor Agonists] explode all trees |
| #5 | (Glucagon Like Peptide 1 Receptor Agonists):ti,ab,kw OR (GLP-1 Receptor Agonists):ti,ab,kw OR (GLP 1 Receptor Agonists):ti,ab,kw OR (Incretin Mimetics):ti,ab,kw OR (GLP-1 Analogs):ti,ab,kw OR (semaglutide) |
| #6 | #4 OR #5 |
| #7 | MeSH descriptor: [Cardiovascular Diseases] explode all trees |
| #8 | (Cardiovascular Diseases):ti,ab,kw OR (Cardiac Events):ti,ab,kw OR (Coronary Artery Disease):ti,ab,kw OR (Major Adverse Cardiac Events):ti,ab,kw OR (mortality):ti,ab,kw OR (Myocardial Infarction):ti,ab,kw OR (Heart Failure):ti,ab,kw OR (Stroke):ti,ab,kw |
| #9 | #7 OR #8 |
| #10 | MeSH descriptor: [Randomized Controlled Trials as Topic] explode all trees |
| #11 | (randomized controlled trial):ti,ab,kw OR (Clinical Trials, Randomized):ti,ab,kw OR (Trials, Randomized Clinical):ti,ab,kw OR (Controlled Clinical Trials, Randomized):ti,ab,kw OR (random*):ti,ab,kw OR (placebo):ti,ab,kw |
| #12 | #10 OR #11 |
| #13 | #3 AND #6 AND #9 AND #12 |

**4.Web of science**

| Search number | Query |
| --- | --- |
| #1 | TS=("Type 2 Diabetes" OR "Diabetes Mellitus, Type 2" OR "Noninsulin-Dependent Diabetes Mellitus" OR "NIDDM" OR "T2DM") |
| #2 | TS=("Glucagon Like Peptide 1 Receptor Agonists" OR "GLP-1 Receptor Agonists" OR "GLP 1 Receptor Agonists" OR "Incretin Mimetics" OR "GLP-1 Analogs" OR " semaglutide") |
| #3 | TS=("Cardiovascular Disease" OR "Cardiac Event" OR "Coronary Artery Disease" OR "Major Adverse Cardiac Events" OR “mortality” OR "Myocardial Infarction" OR "Heart Failure" OR "Stroke") |
| #4 | TS=（“randomized controlled trial” OR “Clinical Trials, Randomized” OR “Trials, Randomized Clinical“ OR ”Controlled Clinical Trials, Randomized” OR “random*” OR “placebo”） |
| #5 | #1 AND #2 AND #3 AND #4 |

**
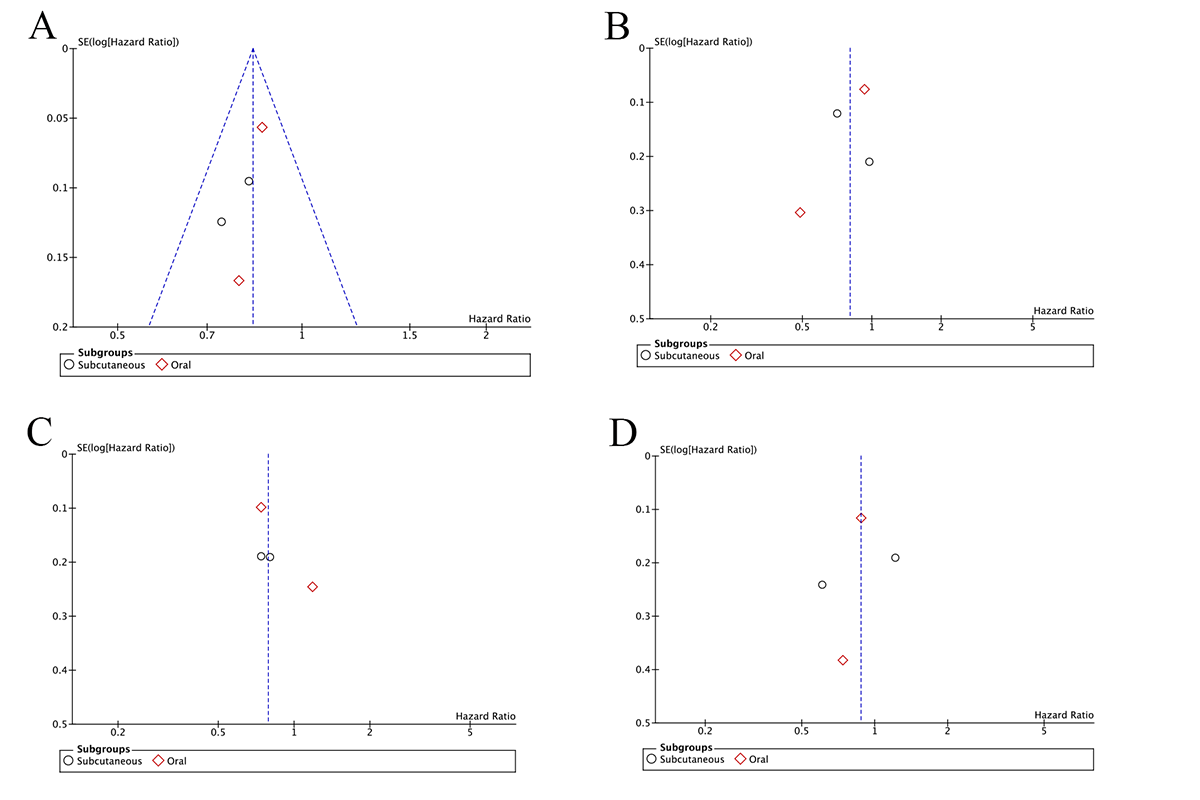
**

**Supplementary Figure S1.** Funnel Plot for Cardiovascular Outcomes: (A) Primary outcome, (B) Cardiovascular death, (C) Nonfatal myocardial infarction, and (D) Nonfatal stroke.

**
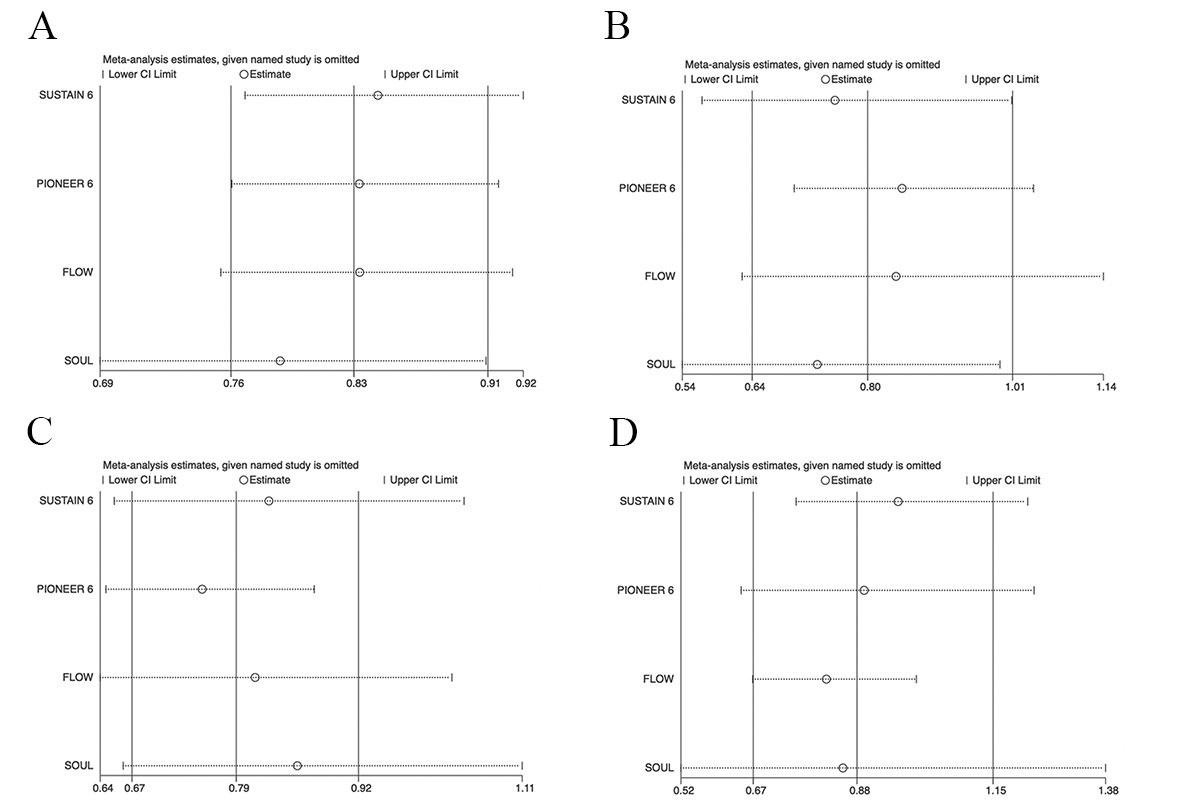
**

**Supplementary Figure S2.** Funnel Plot for Cardiovascular Outcomes: (A) All-cause death, (B) HF hospitalization, (C) UA hospitalization, and (D) Revascularization.

**
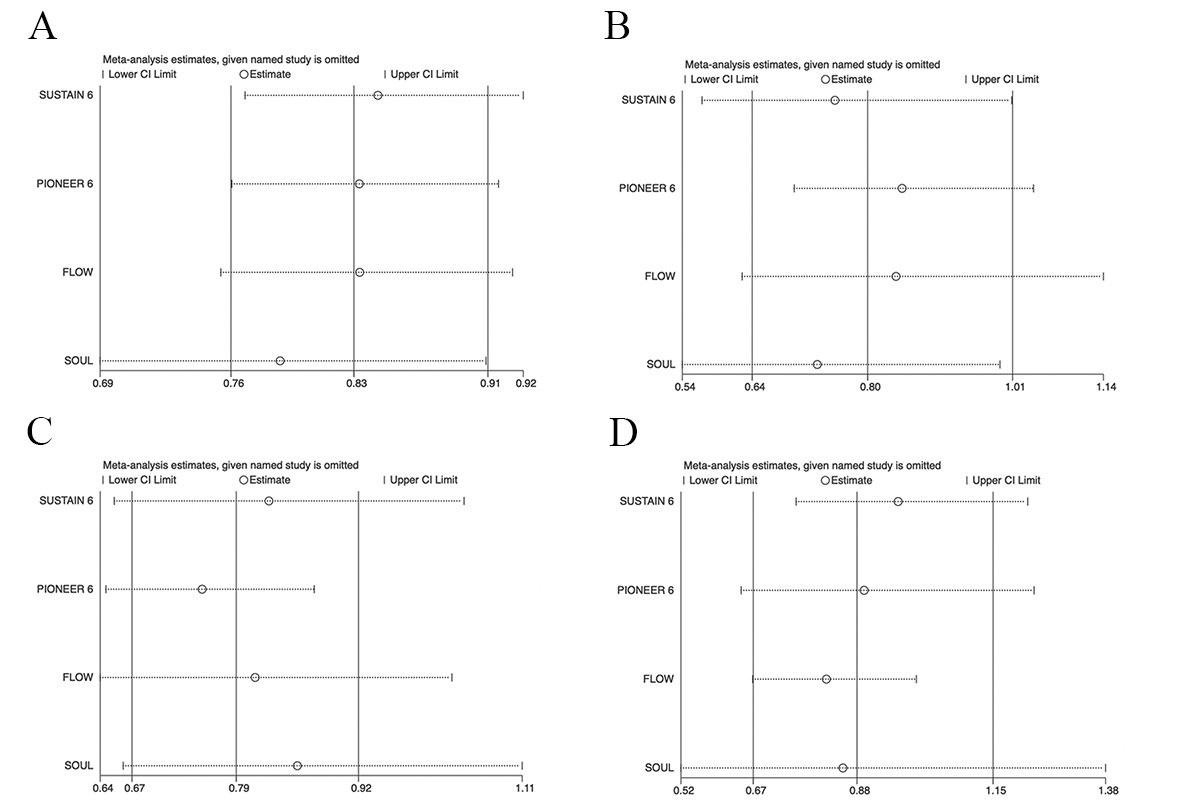
**

**Supplementary Figure S3.** Sensitivity Analysis of Pooled HRs for Cardiovascular Outcomes: (A) Primary outcome, (B) Cardiovascular death, (C) Nonfatal myocardial infarction, and (D) Nonfatal stroke.

**
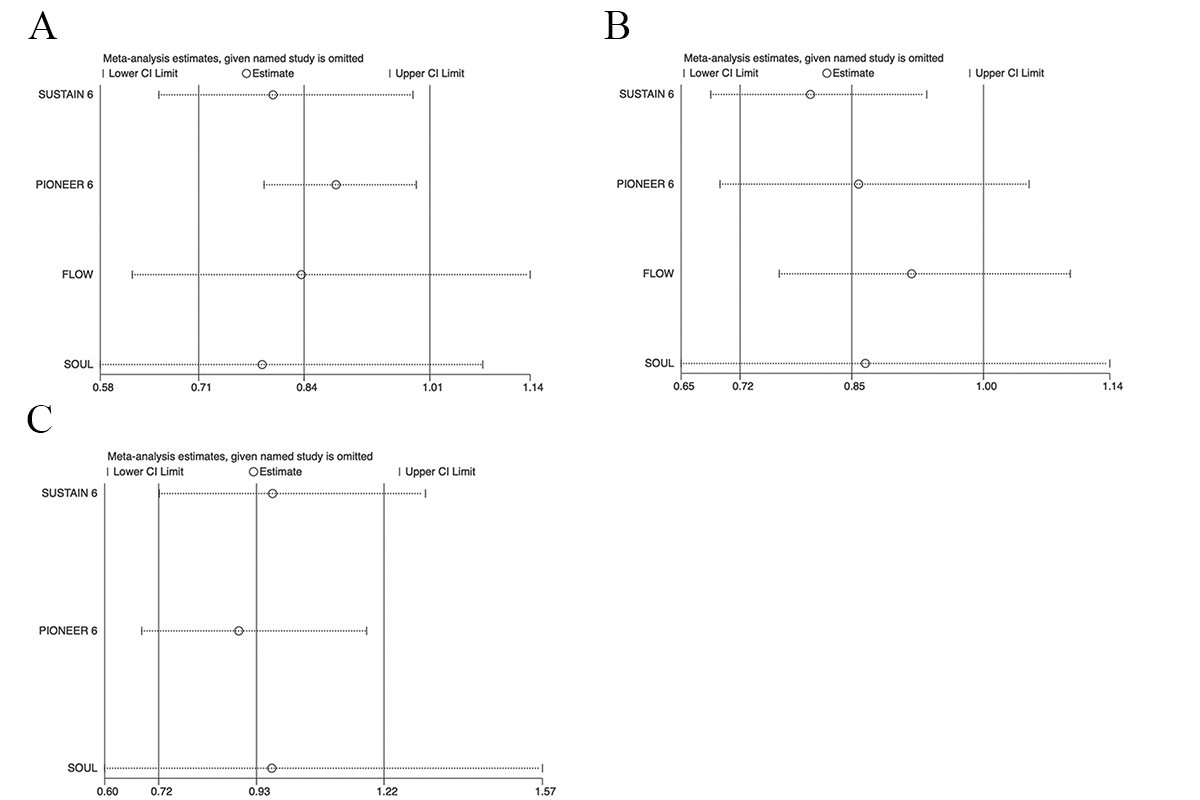
**

**Supplementary Figure S4.** Sensitivity Analysis of Pooled HRs for Cardiovascular Outcomes: (A) All-cause death, (B) HF hospitalization, and (C) UA hospitalization.
